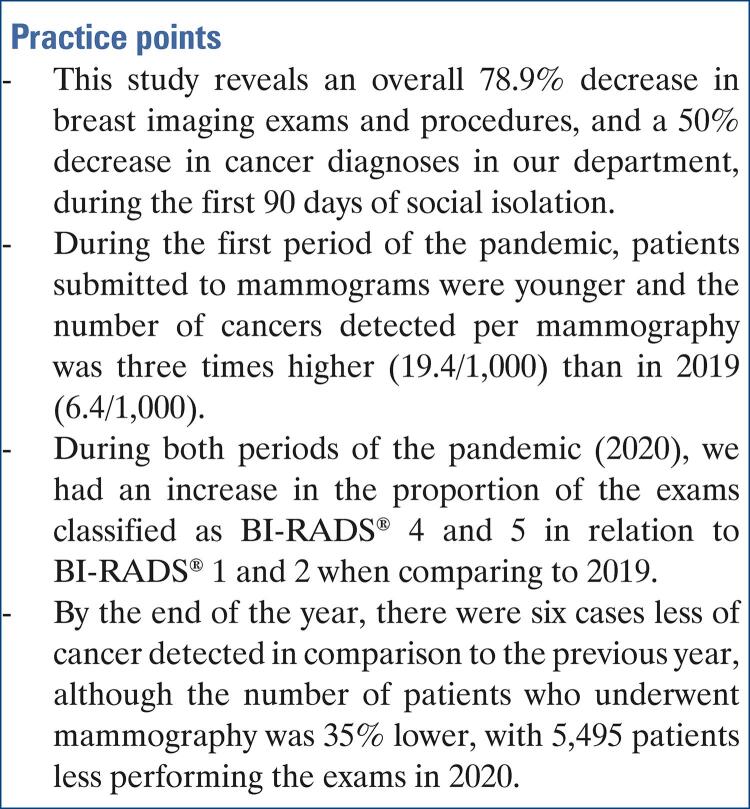# The delay of breast cancer diagnosis during the COVID-19 pandemic in São Paulo, Brazil

**DOI:** 10.31744/einstein_journal/2021AO6721

**Published:** 2021-12-15

**Authors:** Bruna Mayumi Takaki Tachibana, Renato Leme de Moura Ribeiro, Érica Elisangela Françolin Federicci, Renata Feres, Felipe Antonio Sulla Lupinacci, Iviny Yonekura, Ana Claudia Silveira Racy

**Affiliations:** 1 Hospital Israelita Albert Einstein São Paulo SP Brazil Hospital Israelita Albert Einstein, São Paulo, SP, Brazil.

**Keywords:** COVID-19, Coronavirus infections, Breast neoplasms, Early detection of cancer, Mammography, Magnetic resonance spectroscopy, Ultrasonography, mammary, Prognosis, Breast cancer screening, Brazil

## Abstract

**Objective:**

To evaluate the impact of COVID-19 pandemic on breast cancer diagnosis in a breast imaging center.

**Methods:**

This was a retrospective cohort study that included women submitted to breast exams and procedures in a private hospital in São Paulo, SP, Brazil, as from the period of most strict social isolation measures, in 2020 (separated in first period of social isolation, March 24 to June 21, 2020, and second period, June 22 to December 31, 2020), as compared to the same period in 2019. The number of exams, cancer detection rates, pathologic findings and risk factors were analyzed.

**Results:**

A total of 32,144 patients were included in the study. Breast imaging exams and procedures decreased by 78.9% in the first period, and 2.7% in the second period, in 2020. By the end of 2020, the number of breast cancer lesions detected was just six cases less than in 2019, although the number of patients submitted to mammograms was 35% lower.

**Conclusion:**

There was a drop in number of breast exams and cancer diagnoses in the first 90 days of the pandemic. The decrease in diagnosis of cancer was partially compensated in the second period, but the number of patients submitted to mammograms by the end of 2020 was lower, still considering a large number of patients with delayed exams.

## INTRODUCTION

From December 2019 to March 2021, severe acute respiratory syndrome coronavirus 2 (SARS-CoV-2) has caused more than 115 million cases of coronavirus disease 2019 (COVID-19) and over 2.5 million deaths across the world.^( 1 )^ Countries with significant outbreaks, including Brazil, have introduced social distancing or lockdown measures to flatten the curve of incidence of the COVID-19 pandemic, and reduce the potential impact on health care systems. By April 2020, about half of the world’s population was under lockdown, with over 3.9 billion people in more than 90 countries or territories having been asked or ordered to stay at home by their governments.^( 2 , 3 )^

To secure the health of patients and staff and preserve vital resources within the health care system, health care organizations and providers have been instructed to stop performing elective surgical procedures. Furthermore, the American College of Radiology (ACR) has endorsed the guidance from the Centers for Disease Control and Prevention (CDC) to reschedule nonurgent outpatient visits.^( 4 )^ Organizations related to breast cancer diagnosis and treatment also published recommendations for scheduling breast imaging exams and breast cancer treatment during the COVID-19 pandemic. The guidelines were initially to “cancel elective and non-urgent procedures, except those applied to evaluate urgencies, such as abscesses and postoperative complications.”^( 5 )^

Amid the increasing number of COVID-19 cases and similar to other international healthcare providers, the Brazilian *Colégio Brasileiro de Radiologia* (CBR), in association with the Brazilian *Federação Brasileira das Associações de Ginecologia e Obstetrícia* (FEBRASGO) and the *Sociedade Brasileira de Mastologia* (SBM), published a note on March 26, 2020 with recommendations for scheduling breast imaging exams during the COVID-19 pandemic, stating all exams that could be postponed should be avoided, especially in patients aged over 60 years. The advice was very clear addressing all breast exams should be carefully assessed, including screening exams.

In this context, specifically the fear of an unknown and highly transmissible virus, many patients have postponed their breast imaging exams and medical care. There was a drop in all exams, increasing the risk of delayed diagnosis to patients. In July 2020, ACR published recommendations for the cautious recovery and resumption of all types of imaging practices.^( 6 )^

During 2020, there were many fluctuations in the number of cases of COVID-19 and, consequently, in the recommendations for social distance. The population has been waiting for the right moment to return to their usual routine and perform their exams, but much instability has been observed in the number of cases up to the present moment, including new strains of the COVID-19 circulating in the population.^( 7 )^

### History of breast cancer and COVID-19 in our organization

Breast cancer is a very prevalent disease in the world, and it is not different in Brazil. According to data from the Brazilian Ministry of Health and the *Instituto Nacional de Câncer José Alencar Gomes da Silva* (INCA), it was estimated that 66,280 new cases would be diagnosed in Brazil, in 2020. Breast cancer is the major primary cause of death from neoplasms in women in our country, accounting for 16.4% of cases (17,572 deaths per year).^( 8 )^ One of the major public health challenges is to diagnose and treat the cancer as early as possible, increasing the patient’s disease-free survival and life expectancy.

According to the American Cancer Society’s (ACS) biennial update on female breast cancer statistics, the 5-year survival rate is 91%. However, survival decreases greatly if patients develop distant metastases. The overall 5-year relative survival rate is 99% for localized disease, and 86% for regional disease, which drops to 27% for distant- metastasis stage disease.^( 9 )^ A breast cancer diagnosed at a more advanced stage may change from having a curable (with near-normal life expectancy) to incurable disease.

The first registered case of COVID-19 in Brazil was diagnosed at our organization on February 26, 2020, in a patient returning from a trip to Italy. Since then, the number of COVID-19 cases and deaths has increased in Brazil, with more than 16 million people diagnosed and more than 470 thousand deaths by the end of May, 2021.^( 10 )^ The city of São Paulo (SP, Brazil) has 12.2 million inhabitants, and registered over 799 thousand cases and 31 thousand deaths by the same date.

The *Hospital Israelita Albert Einstein* (HIAE) is a quaternary care hospital, with a 579-bed capacity. The imaging department predominantly serves the hospital performing exams in hospitalized and emergency department patients. It also has an outpatient center in charge of the majority of breast imaging exams.

Previous studies about the reduced volume of imaging exams have already demonstrated diminished numbers of breast imaging exams^( 11 - 14 )^ during the pandemic. However, these studies do not make a clear analysis with data by the end of the year, when the population started to receive warnings about the importance of returning to routine exams, and tried to resume their activities with safety precautions for COVID-19.

## OBJECTIVE

To evaluate the impact of COVID-19 pandemic on breast cancer diagnosis in a breast imaging center.

## METHODS

This cohort study included patients from the Imaging Department of HIAE, who underwent breast exams (mammography, magnetic resonance imaging – MRI –, ultrasonography, and invasive procedures) in the first and in the second period of social isolation in our state, respectively, from March 24 to June 21, 2020 and from June 22 to December 31, 2020, compared with the same periods during March 24 to December 31, 2019. The research project was submitted to the Research Ethics Committee of HIAE, protocol 4,321,537, CAAE: 37227920.8.0000.0071. No Informed Consent Forms were obtained. The request for waiver of Informed Consent Form was accepted considering it was retrospective study with anonymized data.

A search for the breast exams was performed using a business intelligence tool and our radiology information system. Data collection included age of patients, the number of newly diagnosed breast cancers, Breast Imaging Reporting and Data System (BI-RADS^®^) final assessment,^( 15 )^ number and results of biopsies, histological and molecular type of the tumors, and risk factors of the patients. Patients diagnosed with malignant breast lesions were classified in symptomatic (presenting with palpable lesions, nipple discharge, nipple retraction and symptoms of metastatic lesions), asymptomatic with increased risk of breast cancer (considering personal or family history including mother, sister, daughter, aunt or first-degree male relatives) and asymptomatic with no increased risk. Information about the symptoms was gathered from the questionnaires that each patient filled out before the procedure. We excluded mammograms with incomplete data in their reports from the BI-RADS^®^ analysis.

Data were presented in absolute and relative frequencies, median and range, and statistical analyses were made using the χ^2^, likelihood ratio, and Student’s *t* tests. The data analysis for this study was generated using the software (SPSS) for Windows, version 26.0 (IBM Corp., Armonk, NY, United States).^( 16 )^

## RESULTS

This study included 32,114 patients from our breast imaging center; in that, 15,888 patients during the pandemic period (March 24 to December 31, 2020) and 23,110 patients during the comparison period (March 24 to December 31, 2019), and 6,884 were seen in both years. A total of 27,215 breast imaging exams (mammography, MRI, ultrasonography, and invasive procedures) were performed at our organization in 2020 (March 24 to December 31), compared with 37,968 exams in 2019 during the same period. Table 1 compares the exams performed during the studied period of 2020 and corresponding period of 2019, categorized by type ( Table 1 ).

**Table 1:** Exams performed during period of 2020 and corresponding period of 2019, categorized by type

Periods	2019	2020	Increase/decrease
First period*			
Mammography	5,844	948	-83.8
Ultrasonography	6,298	1,513	-76.0
MRI	478	141	-70.5
Biopsies	219	105	-52.1
Total	12,839	2,707	-78.9
Second period*			
Mammography	10,379	9,891	-4.7
Ultrasonography	12,970	12,644	-2.5
MRI	1,003	1,051	+4.8
Biopsies	518	613	+18.3
Total	24,870	24,199	-2.7

Results expressed as n or %.

* First period corresponded to the first 90 days of social isolation (March 24 to June 21, 2020) due to COVID-19 pandemic. The second period corresponded to COVID-19 pandemic after the first 90 days of social isolation, by the end of the year (June 22 to December 31, 2020).

MRI: magnetic resonance imaging.

In the first period, we had a significant drop in all exams, more evident in the mammograms. In the second period, the decrease in mammography and ultrasonography was lower than in the first period, and an increase in MRI and biopsies was observed (χ^2^ test, p<0.001).

**Figure 1:**
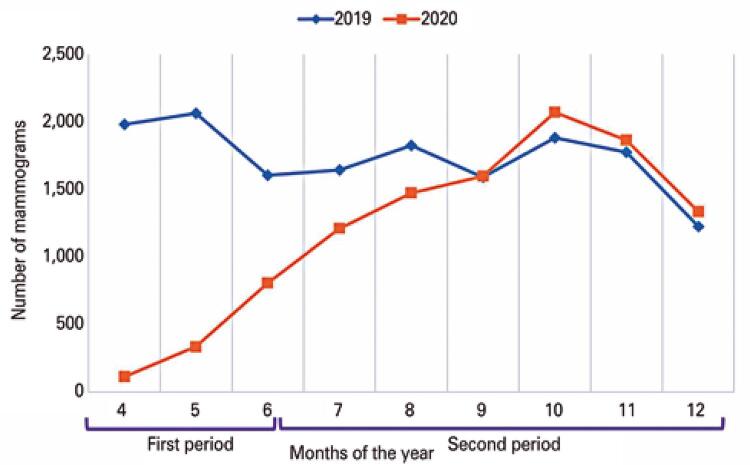
A gradual increase in the number of exams in 2020 is observed, after the sharp reduction in March and April. As from September, the number of mammograms during the pandemic (2020) was higher than in the pre-pandemic period (2019)

The number of mammograms dropped sharply in the first period and gradually returned to baseline parameters, more significantly in May. In September it surpassed the number of exams from the previous year ( Figure 1 ).

From the total of 34,603 mammograms, we excluded 1,525 exams in 2020 and 2,842 in 2019, due to missing data of the BI-RADS^®^ final assessment in our radiology information system.

The mean age of the patients submitted to mammography during the pandemic was lower (50.44) than in the same period of 2019 (52.11), with p<0.001. During the second period, there was no statistically significant difference ( Table 2 ).

**Table 2:** Overall analysis during the first and second periods, March 24 to December 31, 2019 and March 24 to December 31, 2020

Findings	1^**st**^ period			2^**nd**^ period		
	
	2019	2020	p value	2019	2020	p value
Patients submitted to mammography	5,661	927		10,155	9,394	
Age*	52.1±11.24	50.4±10.27	<0.001	51.5±11.04	51.7±10.76	0.218
BI-RADS^®^ 4 and 5^†^	2.7	6.2	<0.001	3.2	3.9	0.014
Breast cancer	36	18		98	110	
Breast cancer/1,000 patients^‡^	6.4	19.4	<0.001	9.7	11.7	0.165
Symptomatic or elevated risk^§^	55.6	88.9	0.016	61.2	60.0	0.857
Aggressive subtypes^¶^	19.4	27.8	0.5	14.3	8.2	0.2

Results expressed as n, mean age±standard deviation or %.

* Mean age of patients submitted to mammography (standard deviation); ^†^ BI-RADS^®^ 4 and 5 among BI-RADS^®^ 1, 2, 3 and 4; ^‡^ rate of breast cancer/patients submitted to mammography; ^§^ percentage of breast cancer in patients presenting symptoms or elevated risk; ^¶^ percentage of more aggressive molecular subtypes (Her-2 and triple-negative).

Considering the mammography BI-RADS^®^ final assessment, we found in the first and second periods of 2020 a decreased percentage of BI-RADS^®^ 1 and 2, and an increase of BI-RADS^®^ 4 and 5, as shown in table 3 .

**Table 3:** Classification of the mammographies as BI-RADS® 1 and 2 and BI-RADS® 4 and 5, separated by year

Period	2019	2020	p value
First period			
BI-RADS^®^ 1 and 2	4,797 (97.3)	693 (93.8)	
BI-RADS^®^ 4 and 5	131 (2.7)	46 (6.2)	
Total	4,928 (100)	739 (100)	<0.001
Second period			
BI-RADS^®^ 1 and 2	8,181 (96.8)	8,239 (96.1)	
BI-RADS^®^ 4 and 5	272 (3.2)	336 (3.9)	
Total	8,453 (100)	8,575 (100)	0.014

Results expressed as n (%).

In 2020, in both periods, we observed an increase in the proportion of exams classified as BI-RADS^®^ 4 and 5 in relation to BI-RADS^®^ 1 and 2 when comparing to 2019. χ^2^ test, p<0.001 (first period)/p=0.014 (second period).

**Figure 2:**
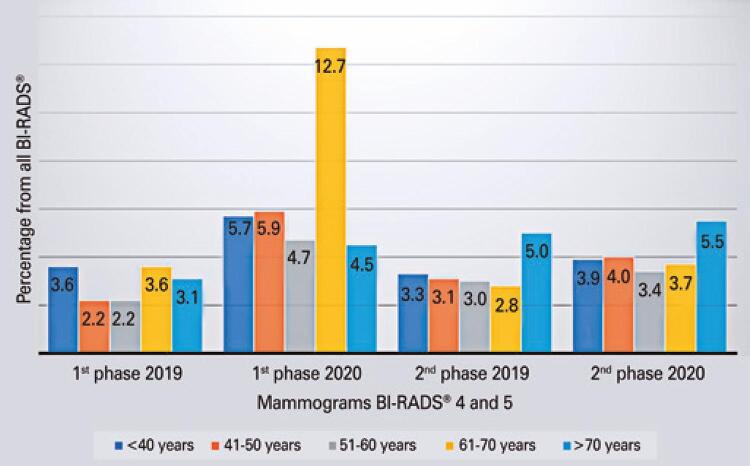
Percentage of mammograms with final assessment BI-RADS® 4 and 5, separated by periods and age groups. An increase in the frequency of exams classified as BI-RADS® 4 in patients aged 61 to 70 years was observed in the first period of 2020, compared to the others (p=0.341)

Among the mammograms with final assessment BI-RADS^®^ 4 and 5, we observed an increased proportion of patients in the age range of 61 to 70 years during the pandemic ( Figure 2 ).

The number of malignant breast lesions diagnosed in 2020 was 130 (18 and 112 in the first and second periods, respectively), and, in 2019, it was 138 (37 and 101 in the first and second periods, respectively). Among the malignant lesions, six cases were not considered as breast cancer (three cases of lymphoma and three cases of methastatic lesions).

A higher rate of breast cancer/1,000 patients submitted to mammograms was observed in 2020, as compared to the same period in 2019 ( Table 4 ).

**Table 4:** Comparison of breast cancer detection / patients submitted to mammography, separated by year (2019 and 2020), during the first (March 24 to June 21) and second (June 22 to December 31) periods

Period of the year	2019			2020			p value
	
	Breast cancer	Number of mammography	Rate/ 1,000	Breast cancer	Number of mammography	Rate/ 1,000	
First period	36	5,661	6.4	18	927	19.4	<0.001
Second period	98	10,155	9.7	110	9,394	11.7	0.165
Both periods	134	15,816	8.5	128	10,321	12.4	0.002

Among the patients diagnosed with breast cancer in the first period of 2020, 88.9% of them had symptoms related to the disease (including palpable lesions, nipple discharge, nipple retraction, and symptoms of metastatic lesions), or increased risk of breast cancer (considering past or family history including mother, sister, daughter, aunt) as compared to 55.6%, in 2019.

Considering the molecular subtypes of breast cancer, there was an increase in the frequency of more aggressive subtypes in the first phase of 2020 (27.8 *versus* 19.4 in the previous year), although with no statistically significant association.

## DISCUSSION

The COVID-19 pandemic has certainly several consequences on the world’s health, many of which have yet to be measured.

This study shows the pandemic caused a global reduction of 78.9% in breast imaging exams and procedures in our department during the first 90 days of social isolation (March 24 to June 21, 2020), as compared to the previous year. The findings of our study are remarkably consistent with the literature, reporting reduced demand for health care during the pandemic, including emergency services during the first months of the pandemic.^( 17 )^ Naidich et al., observed the greatest decline in imaging volume during the pandemic was specifically for outpatient imaging (88%), affecting all modalities – but more pronounced in mammography exams (94% less than 2019).^( 11 )^

In the first period of 2020, the evidence of a higher frequency of BI-RADS^®^ 4 and 5 compared to 1 and 2, and decreased mean age of patients who underwent mammograms, associated with the large increase in the number of breast cancers/1,000 patients, demonstrate, at this stage, the patients who underwent the exams were those who really required them.

This is reinforced when we observe the analysis of malignant lesions, which were more associated to patients with symptoms or increased risk, and more aggressive subtypes than in the previous period (2019).

This finding is in accordance with the study by Al-Thoubaity, which proved the most aggressive subtypes of breast cancer presented with a higher histological grade and larger tumor size upon diagnosis; hence, more likely to be symptomatic.^( 18 )^

The higher frequency of BI-RADS^®^ 4 and 5 mammograms among patients aged 61 to 70 years, in the first period of 2020, can also show most patients in this age group stayed at home, and those who came presented with suspicious findings. Older patients were expected to be affected more with the pandemic restrictions, since social isolation recommendations described this group as high risk for developing more severe clinical pictures of COVID-19 infections.

Based on our findings, we estimated approximately 18 patients from our organization may have had a delayed diagnosis of breast cancer during the first 90 days of the social isolation (a drop by 50% compared to 2019). Similar to our department, public health systems in other countries also have documented a reduction in cancer detection in the first period of the pandemic. The Netherlands Cancer Registry reported a drop in cancer incidence by up to 40% in the last few weeks during the pandemic.^( 19 )^According to the Cancer Research United Kingdom, the number of urgent cancer referrals in England has reduced by 75% since restrictions were implemented.^( 20 )^

In the second period, the increased proportion observed in MRI and biopsies related to mammography and ultrasonography may reflect greater complexity of the cases, associated to the higher rate of cancer/mammograms as compared to 2019.

The number of breast cancer diagnosis in the second period of the pandemic was higher than in the previous year (110 *versus* 98); however, the number of mammograms remained a little lower.

Considering the period March 24 to December 31, the total number of breast cancer in 2020 was just six cases less than in 2019.

The findings of our study suggest there was a delay in the breast cancer diagnosis during the first period of the pandemic, which was partially compensated for in the second.

Nonetheless, we are still concerned about the total number of patients undergoing mammographic examinations remaining 35% below the previous year. Although these may be patients who would have normal exams and would not have malignant diagnoses, if we consider the rate of 8.5 cases/1,000 mammograms in 2019, we estimate 46.7 cases of undiagnosed cancer in 2020 (35.9% of total diagnosed since the beginning of the pandemic).

We have not been able to measure the impact of the pandemic in diagnosis of breast cancer in our population. But some evidence points to a worse prognosis due to late diagnosis. The delay in breast cancer diagnosis probably contributes to more advanced stages upon presentation, leading to poorer clinical outcomes. Maringe et al., in a population-based modelling study, estimated an increase by 7.9% to 9.6% in deaths due to breast cancer up to 5 years after diagnosis, due to delayed diagnosis caused by the pandemic.^( 21 )^

Currently in the pandemic, health systems and facilities are more prepared to receive patients for exams. Hospitals and health facilities are more structured with safety protocols adapted for the prevention of COVID-19 transmission.^( 6 , 21, 22 )^Benefits from breast screening exams outweigh the risk of COVID-19 infection. In this scenario, as breast societies keep advising about the importance of returning to screening exams, we need to do an intense and effective effort so that patients really feel safe enough to return to their breast exams.

Strategies are needed to encourage women to perform postponed breast imaging screening exams, and prepare our team to perform more exams than usual, thus avoiding further delays in diagnosis of breast cancer.

We have to consider the delay in breast exams not only in terms of returning to the usual number of exams, but also addressing how to mitigate the effects of delay in diagnosis.

One limitation of our study was the reduced number of patients and exams during the first period of the pandemic. However, analyzing this reduced number of patients was, in fact, one of the objectives of our work.

To date, this is the first study to analyze the impact of the pandemic in health care of a population seen at a breast cancer diagnostic center, comparing differences in the volume of exams and diagnoses in 2020 and 2019, as from the first days of social isolation until the end of the year, separated into two periods, and assessing the consequences in 2020 due to the pandemic.

## CONCLUSION

The study showed a large drop in the number of breast exams and cancer diagnoses in the first 90 days of the pandemic, with a greater number of patients with suspicious and malignant findings. In the second period, there was a partial compensation of the number of cancer diagnoses and, by the end of the year, the number of breast cancers detected was only six less than in 2019, although the number of patients submitted to mammograms was lower. Future studies may measure the damage caused by the pandemic in treatment and prognosis, secondary to delay of exams and diagnoses.