# Supplementary material for: The delay of breast cancer diagnosis during the COVID-19 pandemic in São Paulo, Brazil
Source: Einstein (Sao Paulo). 2021 Dec 15;19:eAO6721. doi: 10.31744/einstein_journal/2021AO6721 (PMC8687645; doi:10.31744/einstein_journal/2021AO6721)
Supplement: Translation

# Translation: O atraso no diagnóstico do câncer de mama durante a pandemia da COVID-19 em São Paulo, Brasil

**Authors:** Bruna Mayumi Takaki Tachibana, Renato Leme de Moura Ribeiro, Érica Elisangela Françolin Federicci, Renata Feres, Felipe Antonio Sulla Lupinacci, Iviny Yonekura, Ana Claudia Silveira Racy

**Affiliations:** 1 Brasil Hospital Israelita Albert Einstein, São Paulo, SP, Brasil.

**Keywords:** COVID-19, Infecções por coronavírus, Neoplasias da mama, Detecção precoce de câncer, Mamografia, Espectroscopia de ressonância magnética, Ultrassonografia mamária, Prognóstico, Rastreamento de câncer de mama, Brasil

## Abstract

**Objetivo:**

Avaliar o impacto da pandemia da COVID-19 no diagnóstico de câncer de mama em um centro de imagem de mama.

**Métodos:**

Estudo de coorte retrospectivo que incluiu mulheres submetidas a exames e procedimentos de mama em um hospital privado em São Paulo, SP, Brasil, no período de medidas mais rigorosas de isolamento social em 2020 (dividido em primeiro período, de 24 de março a 21 de junho de 2020, e em segundo período, de 22 de junho a 31 de dezembro de 2020), comparado com o mesmo período de 2019. Foram analisados o número de exames, as taxas de detecção de câncer, os achados patológicos e os fatores de risco.

**Resultados:**

Foram incluídas 32.144 pacientes. Os exames e os procedimentos de imagem da mama em 2020 tiveram redução de 78,9% no primeiro período e 2,7% no segundo período. Ao final de 2020, foram diagnosticadas com câncer de mama seis pacientes a menos do que em 2019, embora o número de pacientes submetidas à mamografia tenha sido 35% menor.

**Conclusão:**

Houve queda no número de exames de mama e de diagnósticos de câncer nos primeiros 90 dias da pandemia. A redução dos diagnósticos de câncer foi parcialmente compensada no segundo período, mas o número de pacientes submetidas a exames de mamografia até o final do ano foi menor, considerando ainda um grande número de pacientes com exames atrasados.

## INTRODUÇÃO

De dezembro de 2019 até março de 2021, o coronavírus da síndrome respiratória aguda grave 2 (SARS-CoV-2) causou mais de 115 milhões de casos da doença pelo coronavírus 2019 (COVID-19) e mais de 2,5 milhões de mortes em todo o mundo.^( 1 )^Países com surtos significativos, incluindo o Brasil, introduziram medidas de distanciamento social ou *lockdown* para atenuar a curva de incidência da pandemia da COVID-19 e reduzir o possível impacto nos sistemas de saúde. Em abril de 2020, cerca de metade da população mundial estava sob *lockdown* , com mais de 3,9 bilhões de pessoas em mais de 90 países ou territórios tendo que permanecer em casa, conforme solicitado ou ordenado pelos governos locais.^( 2 , 3 )^

Para garantir a saúde dos pacientes e dos profissionais e preservar recursos vitais dentro do sistema de saúde, instituições e profissionais de saúde foram instruídos a parar de realizar procedimentos cirúrgicos eletivos. Além disso, o *American College of Radiology* (ACR) endossou a orientação dos *Centers for Disease Control and Prevention* (CDC) para que consultas ambulatoriais não urgentes fossem reagendadas.^( 4 )^As entidades atuantes no diagnóstico e no tratamento do câncer de mama também publicaram recomendações para agendamento de exames de imagem da mama e tratamento de câncer de mama durante a pandemia da COVID-19. As diretrizes, inicialmente, recomendavam “cancelar procedimentos eletivos e não urgentes, exceto aqueles destinados a avaliar urgências, como abscessos e complicações pós-operatórias”.^( 5 )^

Tendo em vista o número crescente de casos da COVID-19 e assim como outras entidades médicas internacionais, o Colégio Brasileiro de Radiologia (CBR), em associação com a Federação Brasileira das Associações de Ginecologia e Obstetrícia (FEBRASGO) e a Sociedade Brasileira de Mastologia (SBM), publicou uma nota, em 26 de março de 2020, com recomendações para o agendamento de exames de imagem da mama durante a pandemia da COVID-19, orientando que fossem evitados todos os exames que pudessem ser adiados, principalmente em pacientes acima de 60 anos. A recomendação deixava claro que todos os exames da mama deveriam ser cuidadosamente avaliados, inclusive os de rastreamento.

Nesse contexto específico de medo de um vírus desconhecido e altamente transmissível, muitas pacientes adiaram seus exames de imagem da mama e a atenção médica. Observou-se diminuição em todos os exames, aumentando o risco de atraso no diagnóstico para as pacientes. Em julho de 2020, o ACR publicou recomendações para a recuperação e a retomada cautelosas de todos os tipos de exames de imagem.^( 6 )^

Durante 2020, houve muitas oscilações no número de casos da COVID-19 e, consequentemente, nas recomendações de distanciamento social. A população aguarda pelo momento certo para retornar à sua rotina e realizar exames, mas permanece bastante instável o número de casos até o momento, inclusive com novas cepas da COVID-19 circulantes na população.^( 7 )^

### Histórico do câncer de mama e da COVID-19 em nossa instituição

O câncer de mama é uma doença muito prevalente no mundo e igualmente no Brasil. Segundo dados do Ministério da Saúde e do Instituto Nacional de Câncer José Alencar Gomes da Silva (INCA), estimava-se que 66.280 novos casos seriam diagnosticados no Brasil, em 2020. O câncer da mama é a principal causa primária de morte por câncer em mulheres em nosso país, sendo responsável por 16,4% dos casos (17.572 mortes ao ano).^( 8 )^Um dos maiores desafios de saúde pública é diagnosticar e tratar o câncer o mais cedo possível, aumentando a sobrevida livre da doença e a expectativa de vida das pacientes.

De acordo com a atualização bianual da *American Cancer Society* (ACS) sobre estatísticas do câncer de mama feminino, a taxa de sobrevida em 5 anos é de 91%. No entanto, a sobrevida diminui muito quando as pacientes desenvolvem metástases à distância. A sobrevida relativa global em 5 anos é de 99% para doença localizada e de 86% para doença regional, caindo para 27% para doença com metástase a distância.^( 9 )^Um câncer de mama diagnosticado em estágio mais avançado pode passar de uma doença curável (com expectativa de vida próxima do normal) para incurável.

O primeiro caso registrado da COVID-19 no Brasil foi diagnosticado em nossa instituição em 26 de fevereiro de 2020 em um paciente que retornara de uma viagem à Itália. Desde então, o número de casos e mortes por COVID-19 aumentou no Brasil, com mais de 16 milhões de pessoas diagnosticadas e mais de 470 mil mortes até o fim de maio de 2021.^( 10 )^A cidade de São Paulo, SP, Brasil, com população de 12,2 milhões de habitantes, teve mais de 799 mil casos e 31 mil mortes até essa data.

O Hospital Israelita Albert Einstein (HIAE) é um hospital quaternário, com capacidade de 579 leitos. O Departamento de Imagem atende predominantemente ao hospital, realizando exames em pacientes hospitalizados e de emergência, contando também com um centro ambulatorial responsável pela maioria dos exames de imagem da mama.

Estudos anteriores sobre a redução no volume de exames de imagem já demonstraram números menores de exames de imagem da mama^( 11 - 14 )^durante a pandemia. No entanto, esses estudos não fizeram uma análise clara dos dados até o fim do ano, quando a população passou a receber avisos sobre a importância de retornar a seus exames de rotina e tentava retomar as atividades, de acordo com os protocolos de segurança para a COVID-19.

## OBJETIVO

Avaliar o impacto da pandemia da COVID-19 no diagnóstico de câncer de mama em um centro de imagem de mama.

## MÉTODOS

Este estudo de coorte incluiu pacientes do Departamento de Exames de Imagem do HIAE, submetidas a exames de mama (mamografia, ressonância magnética – RM –, ultrassonografia e procedimentos invasivos) no primeiro e segundo períodos de isolamento social em nosso estado, que foram, respectivamente, de 24 de março a 21 junho de 2020 e de 22 de junho a 31 de dezembro de 2020, em comparação aos mesmos períodos (24 de março a 31 de dezembro) de 2019. O projeto de pesquisa foi enviado para análise pelo Comitê de Ética e Pesquisa do HIAE sob o protocolo 4.321.537, CAAE: 37227920.8.0000.0071. Não foram aplicados Termos de Consentimento Livre e Esclarecido. O pedido de isenção de obrigatoriedade do Termos de Consentimento Livre e Esclarecido foi aceito, por ter sido este um estudo retrospectivo com dados anonimizados.

Foi realizada busca por exames da mama por meio de uma ferramenta de inteligência de negócios, além de nosso sistema de informações de radiologia. A coleta de dados incluiu idade das pacientes, número de câncer de mama recém-diagnosticados, avaliação final do *Breast Imaging Reporting and Data System* (BI-RADS^®^),^( 15 )^número e resultado de biópsias, tipo histológico e molecular dos tumores e fatores de risco das pacientes. Pacientes diagnosticadas com lesões malignas na mama foram classificadas em sintomáticas (que apresentavam lesões palpáveis, derrame papilar, retração de mamilo e sintomas de lesões metastáticas), assintomáticas com risco aumentado de câncer de mama (considerando-se história pregressa ou familiar, incluindo mãe, irmã, filha, tia ou familiares de primeiro grau do sexo masculino) e assintomáticas sem risco aumentado. Informações sobre sintomas foram obtidas dos questionários preenchidos por cada paciente antes do procedimento. Excluímos da análise BI-RADS^®^as mamografias cujos laudos continham dados incompletos.

Os dados foram expressos em frequências absolutas e relativas e em mediana e intervalo, e as análises estatísticas foram feitas usando teste do χ^2^, razão de verossimilhança e *t* de Student. A análise de dados para este estudo foi gerada no programa (SPSS) para Windows, versão 26.0 (IBM Corp., Armonk, Nova Iorque, Estados Unidos).^( 16 )^

## RESULTADOS

Este estudo incluiu 32.114 pacientes de nosso centro de imagem da mama, sendo 15.888 pacientes durante o período da pandemia (24 de março a 31 de dezembro de 2020) e 23.110 pacientes durante o período de comparação (24 de março a 31 de dezembro de 2019), sendo que 6.884 compareceram nos 2 anos. Foram realizados 27.215 exames de imagem da mama (mamografia, RM, ultrassonografia, e procedimentos invasivos) em nossa instituição em 2020 (24 de março a 31 de dezembro), comparados a 37.968 exames no mesmo período de 2019. A tabela 1 compara os exames realizados no período estudado de 2020 e no período correspondente de 2019, categorizados por tipo ( Tabela 1 ).

**Tabela 1:** Exames realizados durante o período de 2020 e o período correspondente de 2019, categorizados por tipo

Período	2019	2020	Aumento/diminuição
Primeiro período*			
Mamografia	5.844	948	-83,8
Ultrassom	6.298	1.513	-76,0
RM	478	141	-70,5
Biópsias	219	105	-52,1
Total	12.839	2.707	-78,9
Segundo período*			
Mamografia	10.379	9.891	-4,7
Ultrassom	12.970	12.644	-2,5
RM	1.003	1.051	+4,8
Biópsias	518	613	+18,3
Total	24.870	24.199	-2,7

Resultados expressos como n ou %.

* Primeiro período correspondeu aos primeiros 90 dias de isolamento social na pandemia da COVID-19 (24 de março a 21 de junho de 2020). O segundo período se deu após os primeiros 90 dias de isolamento social, até o fim do ano (22 de junho a 31 de dezembro de 2020).

RM: ressonância magnética.

No primeiro período, houve queda significativa em todos os exames, sendo esta mais evidente nas mamografias. No segundo período, a queda no número de mamografias e ultrassonografias foi menor do que no primeiro período, observando-se aumento das ressonâncias magnéticas e das biópsias (χ^2^, p<0,001).

O número de mamografias diminuiu acentuadamente no primeiro período e voltou gradualmente aos parâmetros iniciais, mais significativamente em maio. Em setembro, esse número ultrapassou o do ano anterior ( Figura 1 ).

**Figura 1:** Aumento gradual no número de exames em 2020, após acentuada redução em março e abril daquele ano. A partir de setembro, o número de mamografias durante a pandemia, no ano de 2020, foi maior que o do período pré-pandemia (2019)

Do total de 34.603 mamografias, excluímos 1.525 exames em 2020 e 2.842 em 2019, devido à falta de dados sobre a avaliação final do BI-RADS^®^em nosso sistema de informações de radiologia.

A média de idade das pacientes submetidas à mamografia durante a pandemia foi menor (50,44) que no mesmo período de 2019 (52,11), com p<0,001. Durante o segundo período, não houve diferença estatisticamente significativa ( Tabela 2 ).

**Tabela 2:** Análise global no primeiro e segundo períodos (24 de março a 31 de dezembro de 2019 e 24 de março a 31 de dezembro de 2020)

Achados	Primeiro período			Segundo período		
	
	2019	2020	Valor p	2019	2020	Valor p
Pacientes submetidas à mamografia	5.661	927		10.155	9.394	
Idade*	52,1±11,24	50,4±10,27	<0,001	51,5±11,04	51,7±10,76	0,218
BI-RADS^®^4 e 5^†^	2,7	6,2	<0,001	3,2	3,9	0,014
Câncer de mama	36	18		98	110	
Câncer de mama/1.000 pacientes^‡^	6,4	19,4	<0,001	9,7	11,7	0,165
Sintomáticos ou de alto risco^§^	55,6	88,9	0,016	61,2	60,0	0,857
Subtipos mais agressivos^¶^	19,4	27,8	0,5	14,3	8,2	0,2

Resultados expressos como n, média±desvio padrão ou %.

* média de idade das pacientes submetidas à mamografia (desvio-padrão);^†^BI-RADS^®^4 e 5 entre os BI-RADS^®^1, 2, 3 e 4;^‡^taxa de câncer de mama/pacientes submetidas à mamografia;^§^percentagem de câncer de mama em pacientes com sintomas ou alto risco;^¶^percentagem dos subtipos moleculares mais agressivos (Her-2 e triplo-negativo).

Considerando-se a avaliação final BI-RADS^®^para mamografias, encontramos, no primeiro e segundo períodos de 2020, percentagem reduzida de BI-RADS^®^1 e 2 e aumento de BI-RADS^®^4 e 5, como mostra a tabela 3 .

**Tabela 3:** Classificação das mamografias em BI-RADS® 1 e 2 e BI-RADS® 4 e 5, separadas por ano

Período	2019	2020	Valor de p
Primeiro período			
BI-RADS^®^1 e 2	4.797 (97,3)	693 (93,8)	
BI-RADS^®^4 e 5	131 (2,7)	46 (6,2)	
Total	4.928 (100)	739 (100)	<0,001
Segundo período			
BI-RADS^®^1 e 2	8.181 (96,8)	8.239 (96,1)	
BI-RADS^®^4 e 5	272 (3,2)	336 (3,9)	
Total	8.453 (100)	8.575 (100)	0,014

Resultados expressos como n (%).

Em 2020, em ambos os períodos, observamos aumento na proporção dos exames classificados como BI-RADS^®^4 e 5 em relação aos BI-RADS^®^1 e 2, em relação a 2019. Teste χ^2^, p<0,001 (primeiro período)/p=0,014 (segundo período).

Dentre as mamografias com avaliação final BI-RADS^®^4 e 5, observamos uma maior proporção de pacientes na faixa etária de 61 a 70 anos durante a pandemia ( Figura 2 ).

**Figura 2:** Percentagem de mamografias com avaliação final BI-RADS® 4 e 5, separadas por períodos e faixas etárias. Observa-se aumento na frequência de exames classificados como BI-RADS® 4 em pacientes entre 61 e 70 anos no primeiro período de 2020, em comparação aos demais (p=0,341)

O número de lesões malignas da mama diagnosticadas em 2020 foi 130 (18 e 112 no primeiro e segundo períodos, respectivamente), e, em 2019, foi de 138 (37 e 101 no primeiro e segundo períodos, respectivamente). Entre as lesões malignas, seis casos não foram considerados câncer de mama (três casos de linfoma e três casos de lesões metastáticas).

Em 2020, foi observada maior taxa de câncer de mama/1.000 pacientes submetidas a mamografias, em comparação ao mesmo período de 2019 ( Tabela 4 ).

**Tabela 4:** Comparação da detecção de câncer de mama/pacientes submetidas à mamografia, separada por ano (2019 e 2020), durante o primeiro período (24 de março a 21 de junho) e segundo período (22 de junho a 31 de dezembro)

Período	2019			2020			Valor de p
	
	Câncer de mama	Número de mamografia	Taxa/ 1.000	Câncer de mama	Número de mamografia	Taxa/ 1.000	
Primeiro período	36	5.661	6,4	18	927	19,4	<0,001
Segundo período	98	10.155	9,7	110	9.394	11,7	0,165
Ambos os períodos	134	15.816	8,5	128	10.321	12,4	0,002

Entre as pacientes diagnosticadas com câncer de mama no primeiro período de 2020, 88,9% apresentavam sintomas relacionados à doença (incluindo lesões palpáveis, derrame papilar, retração de mamilo e sintomas de lesões metastáticas) ou risco aumentado de câncer de mama (considerando-se história pregressa ou familiar, incluindo mãe, irmã, filha e tia), comparadas a 55,6% em 2019.

Considerando-se os subtipos moleculares de câncer de mama, verificou-se aumento da frequência dos subtipos mais agressivos na primeira fase de 2020 (27,8 *versus* 19,4 no ano anterior), embora sem associação estatisticamente significativa.

## DISCUSSÃO

A pandemia da COVID-19 certamente tem várias consequências para a saúde mundial, e muitas das quais ainda precisam ser mensuradas.

Este estudo mostra que a pandemia causou redução global de 78,9% nos exames de imagem e procedimentos de mama em nosso departamento nos 90 primeiros dias de isolamento social (24 de março a 21 de junho de 2020) em comparação ao ano anterior. Os achados do nosso estudo são bastante condizentes com literatura, com relatos de redução na demanda por cuidados de saúde durante a pandemia, incluindo serviços de emergência, durante os primeiros meses da pandemia.^( 17 )^Naidich et al., observaram que a maior queda no volume de exames de imagem durante a pandemia foi para exames ambulatoriais (88%), o que afeta todas as modalidades – mas, predominantemente, os exames de mamografia (94% menos que em 2019).^( 11 )^

No primeiro período de 2020, as evidências de uma maior frequência de BI-RADS^®^4 e 5 em comparação a BI-RADS^®^1 e 2 e a redução da média de idade das pacientes submetidas a mamografias, associadas ao grande aumento do número de câncer de mama/1.000 pacientes, demonstram que, nesta fase, as pacientes submetidas a exames foram aquelas que realmente precisavam dele.

Isso é reforçado quando observamos a análise das lesões malignas, que foram mais associadas a pacientes com sintomas ou risco aumentado e a subtipos mais agressivos, em comparação ao ano anterior, de 2019.

Esse achado está de acordo com o estudo de Al-Thoubaity, que demonstrou que os subtipos mais agressivos de câncer de mama têm grau histológico mais alto e maior tamanho no momento do diagnóstico, tendo, portanto, mais chance de serem sintomáticos.^( 18 )^

A frequência maior de mamografias BI-RADS^®^4 e 5 entre pacientes de 61 a 70 anos, no primeiro período de 2020, também indica que a maioria das pacientes dessa faixa etária ficou em casa, e as que compareceram apresentaram achados suspeitos. Esperava-se que pacientes mais idosas fossem mais afetadas pelas restrições da pandemia, já que as recomendações de isolamento social descreviam esse grupo como de alto risco para quadros mais graves da COVID-19.

Com base em nossos achados, estimamos que cerca de 18 pacientes de nossa instituição podem ter tido atraso no diagnóstico de câncer de mama nos primeiros 90 dias do isolamento social (redução de 50% em relação a 2019). Semelhante ao nosso departamento, os sistemas de saúde pública de outros países também documentaram redução na detecção do câncer de mama no primeiro período da pandemia. O *Netherlands Cancer Registry* registrou uma queda na incidência de câncer de até 40% nas últimas semanas durante a pandemia.^( 19 )^De acordo com *Cancer Research United Kingdom* , o número de encaminhamentos urgentes por câncer de mama na Inglaterra diminuiu 75% desde a implementação das restrições.^( 20 )^

No segundo período, a maior proporção observada de RMs e biópsias em comparação a mamografias e ultrassonografias pode refletir maior complexidade dos casos, associada à maior taxa de câncer/mamografias em comparação a 2019.

O número de diagnósticos de câncer de mama no segundo período da pandemia foi mais alto que no ano anterior (110 *versus* 98), mas o número de mamografias foi um pouco mais baixo.

Considerando-se o período de 24 de março a 31 de dezembro, o número total de câncer de mama em 2020 foi apenas seis casos a menos que em 2019.

Os achados de nosso estudo sugerem que houve atraso nos diagnósticos de câncer mama durante o primeiro período da pandemia, o que foi parcialmente compensado no segundo período.

No entanto, o que ainda nos preocupa é que o número total de pacientes submetidas à mamografia ficou 35% abaixo do ano anterior. Embora essas possam ser pacientes que teriam exames normais e não seriam diagnosticadas com câncer, se considerarmos a taxa de 8,5 casos/1.000 mamografias de 2019, estimamos 46,7 casos de câncer não diagnosticados em 2020 (35,9% do total diagnosticado desde o início da pandemia).

Ainda não conseguimos medir o impacto da pandemia no diagnóstico de câncer de mama em nossa população. Porém, algumas evidências apontam para um pior prognóstico, devido ao atraso no diagnóstico. O atraso no diagnóstico do câncer de mama provavelmente contribui para uma apresentação em estágios mais avançados, levando a piores desfechos clínicos. Maringe et al., em estudo de modelagem de base populacional, estimaram aumento de 7,9% a 9,6% em mortes por câncer de mama até 5 anos após o diagnóstico, devido ao atraso diagnóstico causado pela pandemia.^( 21 )^

Na fase atual da pandemia, os sistemas e estabelecimentos de saúde estão mais preparados para receber pacientes para exames. Os hospitais e estabelecimentos de saúde estão mais bem estruturados, com protocolos de segurança adaptados para a prevenção da transmissão da COVID-19.^( 6 , 21, 22 )^Os benefícios dos exames de rastreamento do câncer de mama superam o risco de infecção pela COVID-19. Nesse contexto, com as sociedades de mastologia orientando sobre a importância de se retomarem os exames de rastreamento, precisamos de um esforço intensivo e eficaz para que as pacientes se sintam suficientemente seguras para retornarem para seus exames da mama.

São necessárias estratégias para incentivar as mulheres a realizar exames de rastreamento de câncer de mama, além de preparo de nossas equipes para um volume de exames maior que o habitual, evitando atrasos adicionais no diagnóstico do câncer de mama.

Temos que pensar nesse atraso dos exames da mama não somente em termos de como retornar ao número habitual de exames, mas também como mitigar os efeitos do atraso no diagnóstico.

Uma limitação do nosso estudo foi o número limitado de pacientes e exames durante o primeiro período da pandemia. Contudo, analisar esse número limitado de pacientes foi, na verdade, um dos objetivos do nosso trabalho.

Até o momento, este é o primeiro estudo que analisa o impacto da pandemia nos cuidados de saúde de uma população atendida por um centro de diagnóstico de câncer de mama, comparando as diferenças no volume de exames e diagnósticos entre 2020 e 2019, desde os primeiros dias de isolamento social até o fim do ano, separado em dois períodos, além de avaliar as consequências observadas em 2020 devido à pandemia.

## CONCLUSÃO

O estudo mostrou acentuada queda no número de exames e diagnósticos de câncer de mama nos primeiros 90 dias da pandemia, além de maior número de pacientes com achados suspeitos e malignos. No segundo período, houve compensação parcial do número de diagnósticos de câncer e, no fim do ano, o número de câncer de mama detectado foi de apenas seis a menos que em 2019, embora o número de pacientes submetidas a mamografias tenha sido mais baixo. Estudos futuros podem medir os danos causados pela pandemia, no que se refere ao tratamento e ao prognóstico, em decorrência do atraso nos exames e diagnósticos.
